# Something New in Working Plaster of Paris

**Published:** 1870-01

**Authors:** 


### ARTICLE VIII.

### *Something New in Working Plaster of Paris.*

It is a well known fact that powdered gypsum, when freed by calcination of its water of crystallization, regains to a great extent its original hardness when incorporated with water enough to form a stiff paste. In order to attain this end, there is at least thirty-three per cent. of water required wherefrom twenty-two per cent. is withheld as water of crystallization. The rest evaporates, and thus brings about the porosity of the hardened gypsum. In working up a small quantity of gypsum, one has only a few minutes' time for using the paste for moulding or puttying, as it soon becomes hard. With larger quantities, in which case the making of the paste requires a longer time, the mass hardens, sometimes during the operation of dressing. According to Mr. Puscher, of Nuremburg, this inconvenience may be got rid of by mixing with the dry powdered gypsum from two to four per cent. of finely pulverized althea-root and kneading the intimate mixture to paste with forty per cent. of water. In consequence of the great amount of pectin which is contained in the althea-root, and which in fact amounts to about fifty per cent., a mass similar to fat clay is obtained. This mixture begins to harden only after a lapse of one hour's time. Moreover, when dry it may be filed, cut, twined, bored, and thus become of use in the making of domino-stones, dies, brooches, snuff-boxes, and a variety of other things of similar character. Eight per cent. of althea-root, when mixed with pulverized gypsum, retards the hardening for a still longer time, but increases the tenacity of the mass. The latter may be rolled out on window-glass into thin sheets, which never crack in drying, may be easily detached from the glass, and take on a polish readily upon rubbing them. This material, if incorporated with mineral or other paints, and properly kneaded, gives very fine imitations of marble. They bear coloring also when dry, and can then be made water-proof by polishing and varnishing. The artisan in the practice of his trade, will probably find it to his advantage to make use of this prepared gypsum in place of that usually employed by him; the manufacturer of frames need have no fear that wares will crack if he uses a mixture of the above-indicated composition; moreover, the chemist and chemical manufacturer will find that the same does excellent service in luting vessels of every kind. The exact proportion of water to be made use of cannot be given exactly, as it varies within the range of a few per cent., according to the fineness and purity of the gypsum employed. The above-mentioned althea-root need not be of the very best quatity, the ordinary kind serving the purpose perhaps quite as well. —*Druggists' Circular.*

